# “I don’t know if I can keep doing this”: a qualitative investigation of surgeon burnout and opportunities for organization-level improvement

**DOI:** 10.3389/fpubh.2024.1379280

**Published:** 2024-05-10

**Authors:** Kestrel McNeill, Sierra Vaillancourt, Stella Choe, Ilun Yang, Ranil Sonnadara

**Affiliations:** ^1^Department of Psychology, Neuroscience & Behaviour, McMaster University, Hamilton, ON, Canada; ^2^Department of Biology, McMaster University, Hamilton, ON, Canada; ^3^Department of Surgery, McMaster University, Hamilton, ON, Canada; ^4^Department of Surgery, University of Toronto, Toronto, ON, Canada; ^5^Vector Institute for Artificial Intelligence, Toronto, ON, Canada

**Keywords:** burnout, surgery, academic medicine, qualitative methods, payment structure, gender inequity, organizational psychology

## Abstract

**Introduction:**

Burnout is a pressing issue within surgical environments, bearing considerable consequences for both patients and surgeons alike. Given its prevalence and the unique contextual factors within academic surgical departments, it is critical that efforts are dedicated to understanding this issue. Moreover, active involvement of surgeons in these investigations is critical to ensure viability and uptake of potential strategies in their local setting. Thus, the purpose of this study was to explore surgeons’ experiences with burnout and identify strategies to mitigate its drivers at the level of the organization.

**Methods:**

A qualitative case study was conducted by recruiting surgeons for participation in a cross-sectional survey and semi-structured interviews. Data collected were analyzed using reflexive thematic analysis, which was informed by the Areas of Worklife Model.

**Results:**

Overall, 28 unique surgeons participated in this study; 11 surgeons participated in interviews and 22 provided responses through the survey. Significant contributors to burnout identified included difficulties providing adequate care to patients due to limited resources and time available in academic medical centers and the moral injury associated with these challenges. The inequitable remuneration associated with education, administration, and leadership roles as a result of the Fee-For-Service model, as well as issues of gender inequity and the individualistic culture prevalent in surgical specialties were also described as contributing factors. Participants suggested increasing engagement between hospital leadership and staff to ensure surgeons are able to access resources to care for their patients, reforming payment plans and workplace polities to address issues of inequity, and improving workplace social dynamics as strategies for addressing burnout.

**Discussion:**

The high prevalence and negative sequalae of burnout in surgery necessitates the formation of targeted interventions to address this issue. A collaborative approach to developing interventions to improve burnout among surgeons may lead to feasible and sustainable solutions.

## 1 Introduction

### 1.1 Background

Burnout – a psychological syndrome characterized by emotional exhaustion, depersonalization, and a sense of low personal accomplishment– is a significant concern for physicians in North America (1, 2). While prevalence estimates vary, some studies have reported that over 50% of physicians suffer from severe burnout symptoms (3, 4).

Burnout is causing a crisis in healthcare organizations as physicians are experiencing substance misuse, depression, and suicidality at alarming rates (3, 5). Those experiencing burnout report reduced quality of patient care, lower productivity, and are more likely to leave medicine entirely, resulting in significant economic costs (6). Surgeons are at a particularly high risk for burnout due to the high demands associated with their specialty, and have even reported major medical errors in relation to this syndrome (7–9).

Although burnout has been recognized as a consequence of system-level problems, research addressing this issue has primarily focused on individual-focused investigations which have been met with limited success (1, 2, 10). It is critical that we focus our efforts on developing interventions targeted at the source of burnout, which entails examining issues grounded in the workplace. Further, in order to create effective and sustainable interventions, involving stakeholders in the formation of such strategies is critical to ensure feasibility and uptake (11). Leaders in this field of research postulate that examining burnout is more meaningful if it is assessed at the department or unit level as this phenomenon is grounded in the relationships that healthcare workers have within their respective teams (1).

Given the high prevalence of burnout in surgery, it is critical to develop organizational interventions and strategies tailored to surgical departments to promote sustained and meaningful change at the institutional level (1). Our investigations should be targeted accordingly and center techniques which are able to give voice to those most impacted by burnout (11). Qualitative methods present as an ideal way to explore characteristics of work environments contributing to burnout due to their ability to capture the nuance and complexity of different phenomena that cannot be explained using traditional quantitative methods. Moreover, the strength of qualitative research lies in its ability to center participant experiences and translate their perspectives into tangible change.

The majority of burnout research that has been conducted to investigate drivers of burnout in surgical settings has been quantitative in nature and situated across different subspecialties, departments, and institutions (8). These studies tend to be focused on producing generalizable findings applicable to healthcare professions and burnout as a whole or center their analyses on individual-level risk factors (8, 12). While this research has formed the foundation for what we know about who is impacted by burnout in surgery, these methods neglect the value of examining context-specific social dynamics and the role of the organization in the emergence of this syndrome. Furthermore, the voices of surgeons are not fully represented by the quantitative metrics and outcome measures typically employed in these studies. We propose that qualitative methods embedded within an organizational lens are more appropriate for generating insights and interventions tailored to surgeons’ experiences within specific settings.

Thus, the aim of this current research was to employ a qualitative case study methodology to identify the drivers of burnout among surgeons at a single academic medical center using an established framework for organizational evaluation, the Areas of Worklife (AoW) Model (13, 14). This model was developed to point to contributors to burnout that can be translated into organizational strategies for change and identifies six key areas which contribute to burnout. Ultimately, this framework was used to investigate the following question:

*How do surgeons at McMaster University describe their experiences with burnout and what strategies do they suggest might mitigate the emergence of burnout in this context?*

The Department of Surgery at McMaster University is an academic training center affiliated with the Hamilton Health Sciences and St. Joseph’s Healthcare Hamilton hospital networks. Both networks operate within the context of the publicly funded, privately operated Ontario healthcare system, meaning the hospital networks receive funds from the Ontario Ministry of Health, but are independently managed by hospital executives (15). There are 194 surgeons in the department divided among 11 divisions. The staff in the department provide care in various locations, including a major trauma center at Hamilton General Hospital and community hospitals outside of the academic training centers.

All affiliated surgeons are expected to contribute to teaching, research, or program administration in a capacity of their choosing. Most surgeons within the department are compensated through the fee-for-service model, which is the most common compensation plan for physicians in Ontario (16). Under this payment plan, surgeons perform a service for their patients, and subsequently bill the Ontario Ministry of Health for that service. They are then reimbursed a predetermined amount based on the billing code of the service, such as a consultation, procedure, or follow-up visit. In this way, surgeons are paid a discrete amount per service they provide. Alternatives to the fee-for-service model include salary-based compensation, in which institutions receive lump sum payments from the Ministry of Health that are then allocated to physicians according to their contracts (17). Capitation models involve billing the Ministry of Health based on the number of patients under a physician’s care rather than the services provided and are most common in family medicine (18). Salary and capitation payment models are examples of alternative funding plans (AFPs). AFPs made up approximately 27.4% of the total clinical payments to Canadian physicians in 2018, with fee-for-service payments making up the remaining 72.6% of payments (19). These models are not mutually exclusive, and many physicians will be reimbursed through some combination of the two (19).

Compared to the rest of Canada, Ontario has the lowest per-capita healthcare spending rate (20, 21). The province is also facing a nursing shortage, with approximately 23,000 nurses fewer than the national *per capita* average and many considering leaving the profession entirely (22–24). These factors, among others, have contributed to the health care crisis in Ontario that has ultimately resulted in over 1,200 vital service closures at public hospitals in 2023 (25, 26).

In response to the COVID-19 pandemic, the Ontario Ministry of Health (along with public health agencies across Canada) placed restrictions on non-essential surgeries in an attempt to conserve hospital resources and limit the transmission of the virus (27). During the first two and a half years of the pandemic, approximately 13% fewer surgeries were performed in Canada, increasing the already long surgical wait lists across the country, with wait times often exceeding what specialists consider to be clinically “reasonable” (28, 29). Amid this crisis, various calls to action have highlighted the importance of addressing physician burnout to ensure that there are sufficient human resources available to address the system-level issues exacerbated by the pandemic, including the backlog of surgical procedures, and build a more sustainable healthcare system (30).

### 1.2 Philosophical and theoretical foundations

This study was approached from a critical realist lens, which is conceptualized as being ontologically realist and epistemologically relativist. The critical realist position postulates that while an independent reality exists (ontological realism), our representations and thoughts regarding reality are influenced and mediated by cultural and social contexts (epistemological relativism) (31). Essentially, while a reality exists external to the human mind, different methods of study will produce different representations of such reality. For the purposes of this research, it was assumed that there are common drivers of burnout among surgeons within the department, and the responses obtained from the interviews and survey data reflect the unique experiences and understandings of surgeons with these drivers. Moreover, the themes constructed from this data are also reflective of the researchers’ interpretation of the data and the experiences, knowledge, and understanding they bring to the analysis.

Central to the interpretation of this data was the AoW model. The AoW model was created by Maslach and Leiter as a way to identify the key stressors that exist within organizations and develop strategies to improve the job environment (13, 14, 32). This model conceptualizes burnout as being a result of a lack of “fit” or “match” between six different areas pertaining to the workplace: workload, control, reward, community, fairness, and values (Table 1). The greater the incongruence between the person and the environment within each domain, the greater the likelihood the worker is experiencing burnout. In contrast, when high levels of fit are achieved, workers are more likely to be resilient to conditions of stress and engaged with their work (11). Identifying areas from this model in which conditions can be improved (i.e., low levels of fit) can lead to the development of strategies targeting issues at their source and effectively ameliorate burnout (10). To ensure congruence between data collection and the theoretical foundations of this study, the questions posed to participants were framed around the six Areas of Worklife to identify specific problem areas within each domain (Supplementary material).

**Table 1:** An overview of the areas of worklife model.

Area of Worklife	Description
Workload	Lack of fit: A chronic workload that exceeds individuals’ capacity to meet their professional demands without an opportunity to recover from strenuous tasks/periods of time. Fit: A sustainable workload that provides professional opportunities to use and grow skills while providing opportunities for rest.
Control	Lack of fit: Insufficient opportunity to make decisions around the nature of one’s work, including shaping the environment to meet the demands of the job. This includes conflicting or ambiguous requirements in the workplace. Fit: Being able to exercise professional autonomy, including the capacity to influence decisions that impact one’s work (such as gaining access to resources necessary for the job).
Reward	Lack of fit: A deficiency of monetary, social, and intrinsic reward that is incongruent with the expectations one has, leading to the devaluation of the work and the worker themselves. Fit: Feeling consistently and adequately rewarded/recognized for the contributions made in the workplace.
Community	Lack of fit: Ongoing working relationships characterized by unresolved conflict and a lack of support. Fit: A community of workers exists that fosters a sense of inclusion and mutual support/respect.
Fairness	Lack of fit: Decisions at work are perceived as being unfair or inequitable, including promotional, compensation, or procedural-based decisions. Fit: Evaluations, resource distribution, and compensation are perceived as fair and equitable. Leadership as a whole is seen as valuing fairness.
Values	Lack of fit: A gap between the organization and individual values, including a discrepancy between stated organizational values and actual practices or personal morals. Fit: The work people perform in their organization is congruent with their values and ideals, with the individual’s original attraction to the job.

Leiter and Maslach (13, 14).

## 2 Materials and methods

### 2.1 Design and data collection

An intrinsic case study design was chosen for this study as this methodology is ideal for examining issues that are context-dependent, and allows the researcher to focus on the phenomenon of interest while being situated in a particular social and physical context (33, 34). The “case” or unit of analysis under study for this research was the Department of Surgery at McMaster University. Data collection was limited to surgeons within the department, and the analysis focused on elucidating issues specific to this group of physicians.

The qualitative data collected for this study was obtained through a cross-sectional survey and one-on-one interviews that were approximately one hour in length. Surgeons were recruited to participate in the study through a mixture of direct emails and advertisements distributed by the department newsletter. Within the emails and newsletter contained a link to the survey, which collected demographic information, open-ended response questions, and an option to indicate whether the participant was interested in completing a one-on-one interview over video conferencing. Burnout scores were also obtained within the survey using the Maslach Burnout Inventory (MBI), specifically the Human Services Survey for Medical Personnel version (15). This scale produces a score for each of the defined symptoms for medical personnel, being emotional exhaustion, depersonalization, and personal accomplishment using items such as, “I feel emotionally drained from my work,” “I have accomplished many worthwhile things in this job,” and “I do not really care what happens to some patients.” The symptoms can emerge in varying degrees in response to different stressors, with burnout being considered a complex, continuous construct rather than a dichotomous variable. However, cut-offs have been proposed to distinguish severe burnout symptoms (high emotional exhaustion (≥27) with either high depersonalization (≥13) or low personal accomplishment (≤33) (16)), which were used to characterize our sample for descriptive purposes only.

Prior to the formal implementation of the survey and interviews, the data collection form and interview guide were piloted with three surgical faculty members to ensure coherence and clarity (Supplementary Material). Qualitative questions were provided within the survey to facilitate the collection of data from a very time-restricted population in an accessible manner. This method would allow surgeons to provide information without the burden of scheduling a dedicated interview time, and afford participants control over when, where, and how they answered our questions (35). While depth of data is often lost in qualitative surveys, we felt as though the sensitive nature of this topic might have inhibited participants from sharing certain workplace experiences in traditional “face-to-face” data collection; the level of anonymity provided by this survey would potentially facilitate disclosure and ameliorate concerns regarding identification.

Being mindful of participant time restraints and the potential for disengagement, we decided to limit the qualitative portion of the survey to four broad questions. With the additional demographic information collected in the survey, our piloting indicated that the questions would take approximately 15 min to complete (as opposed to an hour-long interview) – a survey length which has maximized participant engagement in past studies conducted within this population. Moreover, for qualitative surveys which focus on lived experience and seek detailed responses, past research has indicated that a small number of questions is ideal (35).

In contrast, the interview guide was constructed with the intent of gathering detailed information pertaining to surgeons’ lived experienced within the scope of the AoW model, and consisted of questions targeting each domain. Field notes were maintained throughout the interviews to capture contextual details and insights during the data collection process. Specifically, information was captured about the location in which the interview was taking place for the participants, their emotional state, and reactions the interviewer had to the information gathered. All interviews were audio recorded and transcribed verbatim.

Interviews were limited to a virtual medium given the COVID-19 related restrictions in place for in-person research. However, this medium also allowed surgeons to choose a convenient time and location in which they completed the interview, and access to a geographically dispersed demographic. Virtual interviews have demonstrated significant emulation of natural conversation, and the ability to elicit data with substantial richness, similar to that of in-person methods (36, 37). While this method offered various advantages, the virtual “context” does limit researchers’ impressions of the physical environment in which the interview is conducted (37, 38). Moreover, the digital medium introduces new contextual factors such as internet connectivity, device preferences, and familiarity with the online platform employed. It should also be noted that online interviews allow participants to curate the appearance of their environment, which may have influenced researchers’ perceptions of surgeons’ contexts (38). Nonetheless, virtual interviews offer access to spaces that may not be readily accessible in traditional face-to-face interviews, and the opportunity to observe unintended disruptions. These considerations were taken into account during the data collection process.

Recruitment was evaluated on an ongoing basis and was informed by the concept of information power, whereby the sample’s adequacy was judged based on the relevancy of the data collected, specificity of participant experiences, quality of dialog obtained, the theoretical background of this study, and the exploratory analytical strategy (39). Data collection ceased when researchers determined that a comprehensive interpretive analysis was achieved and allowed for credible conclusions that were consistent with the research question.

### 2.2 Ethical considerations

This study was granted ethics approval by Hamilton integrated Research Ethics Board (Study #13561). Consent was obtained through standard implied consent language within the survey’s preamble, and verbal consent was again obtained over video conferencing for interviews. Given the established issue of work-related burden and burnout in surgical environments, we endeavored to limit the burden of our data collection on participants by restricting the number of questions in our survey and limiting interviews to being an hour in length. During the interviews, participants were also monitored for behavioral signs of emotional distress and were offered breaks and opportunities to pause or cease the interview if these were noted by the interviewer. Participants were also provided a list of resources and services that they would be able to access in the case of distress, both before and after the interview as well as the survey questions.

To ensure privacy and anonymity, participants were encouraged to take the virtual interviews in place where they would be most comfortable talking about workplace-related issues. We did not impose any restrictions on where this might be, and maintained flexible schedules to accommodate surgeons’ working hours so they were able to take these meetings in a private location, including private hospital, university, and home offices. The option to exclusively provide survey responses was included in case participants did not feel comfortable disclosing their identities to investigators. In terms of monitoring the potential distress of those collecting the data, the interviewers maintained dedicated debriefing times with one another to discuss any potential concerns they had about their own wellbeing, or participant distress. The lead interviewer (KM) also held regular check-in meetings with the co-PIs (RS & IY) to debrief interview experiences and concerns regarding data collection.

### 2.3 Analysis

Braun and Clark’s (31, 40) approach to reflexive thematic analysis was used to analyze the data collected in both the survey and interviews. This approach was also chosen since it is a theoretically flexible technique to theme development, and suits questions exploring people’s experiences, perceptions, and representations of a given phenomenon. Thus, reflexive thematic analysis was an appropriate method to explore surgeons’ experiences with burnout using the AoW model. Specifically, an experiential approach to data analysis was employed, during which the meanings and experiences provided by participants were centered during coding. This analytical method’s flexibility also meant that our analysis could focus on constructing codes which encompassed both semantic (explicit) and latent (implied) meaning in the data and involve inductive (data-driven) as well as deductive (theory-informed) theme development (31). Essentially, while the analysis was centered on developing codes around surgeons’ experiences and interpretations of burnout based on the AoW model, coding also captured issues related to the burnout experience outside of the AoW model. Codes were generated relative to our conceptual framework based on researchers’ theoretically informed interpretation of the data in conjunction with inductive codes that were reflective of researchers’ understanding of participants’ experiences (that may have been unrelated to the AoW model).

Three researchers (KM, SV, SC) followed the six iterative steps of (1) data familiarization, (2) generating initial codes, (3) generating themes, (4) reviewing potential themes, (5) defining and naming themes, and (6) producing the thematic report. It should be noted that this process was iterative and recursive, with coders moving back and forth through the phases as necessary. Within this approach, it is expected that new insights may arise during theme development which may require further iterations of earlier phases and new interpretations of the data (41). The coders held weekly meetings to discuss insights generated throughout the analytical process, collaboratively construct meaning from the data, and promote reflexivity through conversations around the researchers’ assumptions, beliefs, and backgrounds as they related to this study. Interviews were coded in Microsoft Word using the comment function to tag the data with code labels. The software R was then used to extract the comments along with the tagged data excerpts and place them into an excel spreadsheet along with the coded survey data. The codes were refined by collapsing those that shared similar underlying concepts, and eventually formed into themes and subthemes representative of the overarching relationships between codes. After an initial round of themes were developed, these themes were reviewed in relation to the broader dataset, finalized codes, and research question to ensure the interpretations and information provided addressed the original intent of the study. Themes were then defined, named, and reported collaboratively to produce a coherent narrative of the data. Quotes are provided alongside the relevant theme, with details regarding the quote’s origin (S=Survey, I=Interview), the participants gender (M = Man, W=Woman), and whether they hold a leadership position in the department concerning research, education, or administration (L = Leadership). Participants’ characteristics were summarized using descriptive statistics, with means, standard deviations (SD), and frequencies being presented where appropriate.

## 3 Results

### 3.1 Participant characteristics

A total of 28 unique surgeons from the department participated in this study-22 surgeons provided qualitative comments throughout the survey, while 11 participated in one-on-one interviews. In terms of burnout, 18 (64.3%) participants were classified as suffering from severe symptoms, with the average emotional exhaustion, depersonalization, and personal accomplishment scores being 30.4 (SD: 10.8), 10.3 (SD: 10.3), and 37.5 (SD: 6.9), respectively. Of those that provided qualitative survey comments, 9 (40.9%) held leadership positions in the department, and 8 (36.4%) were women while the remaining 14 (63.6%) were men. The average time on faculty for these participants was 15.5 years (SD: 11.0 years). The surgeons that participated in the interviews were composed of 6 (54.5%) men and 5 (45.5%) women; the majority (*n* = 7, 63.6%) held leadership roles in the department, and the average time on faculty was 20.7 years (SD: 11.0 years). On average, participants indicated that just over 65% of their time is dedicated to clinical activities, with their remaining time being distributed across education (mean: 13.1%), research (mean: 9.4%), and administrative work (mean 12.4%). An overview of these characteristics is available in Table 2.

**Table 2:** Participant demographic characteristics.

	Total participants (*n* = 28)*	Survey participants (*n* = 22)	Interview participants (*n* = 11)
Gender, *n* (%)			
Men	18 (64.3%)	14 (63.6%)	6 (54.5%)
Women	10 (35.7%)	8 (36.4%)	5 (45.5%)
Years in independent practice, mean (SD)	17.4 (10.3)	16.4 (10.2)	21.5 (9.4)
Years on faculty, mean (SD)	15.5 (11.0)	15.5 (11.0)	20.7 (11.0)
Leadership position, *n* (%)	15 (53.6%)	9 (40.9%)	7 (63.6%)
Pay structure, *n* (%)			
Fee-for-service	23 (82.1%)	20 (90.9%)	7 (63.6%)
Alternative payment plan	5 (17.9%)	2 (9.1%)	4 (36.4%)
% Time breakdown, mean (SD)			
Clinical	65.2 (17.9)	70.9 (12.6)	55.9 (19.7)
Research	9.4 (11.6)	8.9 (9.1)	8.8 (13.9)
Administration	12.4 (15.1)	9.0 (6.7)	18.8 (22.0)
Education	13.1 (9.4)	11.2 (6.6)	16.5 (11.0)
Burnout			
Severe symptoms, *n* (%)	18 (64.3%)	16 (72.7%)	6 (54.5%)
Emotional exhaustion, mean (SD)	30.4 (10.8)	31.0 (10.6)	29.5 (11.2)
Depersonalization, mean (SD)	10.3 (6.0)	11.1 (5.7)	9.1 (5.4)
Personal accomplishment, mean (SD)	37.5 (6.9)	35.7 (6.4)	41.1 (5.6)

*A total of 28 unique surgeons participated in this study. Three surgeons provided survey responses and opted to complete a virtual interview.

### 3.2 Themes

#### 3.2.1 The Red Queen’s Race: moral injury and chronic overwhelm in the face of high patient volume

While surgeons discussed the impact of a highly demanding schedule on their wellbeing, the main concerns expressed were not related to the nature of clinical work itself, but rather the moral injury associated with being unable to treat patients in the thorough and timely manner that surgeons value and patients require. Surgeons asserted that it is not the number of patients they are treating that threatens to overwhelm them, but rather the number of patients that they *cannot* treat; it was stated that surgeons want more time in the operating room, and struggle immensely with being unable to provide timely care for their patients. The long wait times encountered by patients and the blame surgeons receive (from others and themselves) for these wait times are major sources of stress for physicians. One participant shared the toll that turning patients away has on them by saying,

*“Hundreds and hundreds of patients out there in that catchment area get a letter saying that there’s no doctor to see you. That just feels terrible. [...] I do not have the resources to take care of them,” [004-I-W-L].*

The seemingly infinite flow of patients prevents surgeons from feeling the satisfaction associated with completing a case as there are hundreds more waiting next in line, with many such patients waiting beyond ideal timelines. When asked what aspects of their work contributed to burnout, one participant replied,

“*Sometimes it’s just the never-ending-ness of it. You know, you think if you see these people, you get these emergency referrals and everything else and you see them, and then, you know, there are still more the next week and the next week and the week after that, so it’s as if you are never going to get anywhere,” [006-I-M-L].*

Participants conveyed that it is difficult to feel accomplished in their work when they lack the resources to adequately look after patients on their waitlists. The stress and guilt are worsened by the need to make critical decisions regarding which patients need to be seen first. The surgeons interviewed expressed the concern that longer wait times may exacerbate patients’ conditions and described extreme pressure to triage patients effectively. Participants shared that they live with the constant fear of being sued for inaccurately assessing a patient’s need for care and indirectly contributing to poor outcomes. While legal concerns were indicated explicitly, struggles with moral injury were revealed implicitly. For surgeons in this context, moral injury was outlined in reference to surgeons’ inability to provide timely care to their patients and the subsequent feelings of guilt associated with these delays. Surgeons shared that they entered the field because of their desire to help people, and the constraints placed by time, resources, and politics contribute to major feelings of guilt, inadequacy, and insecurity- prominent emotions associated with moral injury.

One surgeon described the department of surgery as “*A very resource restricted environment*” as a result of “*ongoing cutbacks, especially with the ward and O.R. [operating room] resources, combined with a lot of disparity and inequity in utilization,*” *[009-I-M]*. Surgery was described as a specialty that is heavily reliant on nursing and allied health professionals, other hospital staff, and anesthesiologists, meaning that issues outside of the surgical department can have major ramifications for surgeons. It was provided that staff shortages prevent surgeons from doing their jobs, and this lack of control creates frustration. One participant expressed this concern by saying, “*There are still certain resources, like the procedure room in particular, that [are] very unpredictable,*” *[010-I-W-L]*. This unpredictability creates stress in surgeons’ professional and personal lives as last-minute accommodations and schedule changes can strain relationships with patients as well as with a surgeons’ family and friends.

Many of the surgeons interviewed feel the burden of the failures of the healthcare system falling onto their shoulders, leading to cynicism and exhaustion that are characteristic of burnout. These system-level issues are largely out of a surgeon’s control, yet they impact surgeons’ ability to work in a way that aligns with their values. One surgeon expressed this difficulty in saying,

“*It feels so discouraging because, you know, so much of whether or not they are [patients] going to get better is outside of my control,*” *[010-I-W-L]*.

Ultimately, high patient volumes contribute to burnout in surgeons due to a relative lack of resources available for surgeons to care for those patients. Barriers to O.R. access deprive surgeons of fulfillment in their job and create anxiety that delays in patient care may compromise care. Many surgeons gravitate toward the profession because of the sense of agency it offers in directly making a difference in their patients’ lives. Consequently, keeping patients out of arms’ reach creates guilt and frustration that inevitability intensifies burnout.

#### 3.2.2 No good deed goes unpunished: the role of recognition and remuneration

Reward was identified as another AoW that is relevant to surgeons’ burnout, specifically in relation to compensation models and the downstream impacts on surgeons’ motivation to complete certain responsibilities. Surgeons expressed concern regarding the structure of the fee-for-service compensation model, as well as frustration with a lack of support and recognition for service in leadership and administrative roles.

At academic centers such as McMaster University, surgeons may receive stipends for involvement in research, education, and leadership in addition to compensation for their billed procedures, but participants in the present study highlighted that the reimbursement per hour for these activities is far less than the income that would be made via clinical work. Participants expressed that this payment plan rewards individuals who complete the most procedures, and indirectly disadvantages individuals who take on more time-consuming cases, have greater involvement in research, education or leadership, and complete services that have been determined to be of lower value by the Ministry of Health.

Participants highlighted that because of this model, surgeons are often not directly compensated for the administrative work that they complete. Surgeons described patient care as the most fulfilling aspect of their practice, while administrative tasks such as charting, the clerical burden imposed by electronic health records, and responding to referrals were described as less enjoyable. Administrative aspects of education-based work, such as completing forms related to entrustable professional activities as part of competency based medical education and scheduling meetings for various service-based positions were also provided as sources of this burden. This discrepancy in compensation for the time dedicated to certain tasks results in a large conflict from the perspective of the AoW model, as surgeons are not receiving equitable intrinsic or extrinsic reward (such as fulfillment and compensation, respectively) for completing this labor. This conflict threatens to cause frustration and exhaustion among surgeons, as administrative work becomes a barrier to the sense of fulfillment that patient care provides and is often not directly or appropriately compensated financially.

Other roles within the department, such as leadership, education, and research positions, also tend to not be compensated to the same extent as clinical work. Participants stated that when surgeons choose to take on these roles, they are forced to reduce the time they dedicate to clinical tasks and decrease overall earnings, or else complete their non-clinical commitments in their time off, contributing to the negative impact of overwork that was explored in the previous theme. Participants indicated that as a result, they feel as though they are punished for looking out for the good of the department. One participant described this financial tension between clinical and non-clinical work in saying,

“*The fee schedule is set up that if you spend an hour doing research or education… if you’d spent that hour doing clinical work, the financial reward would be 10 fold or 20 fold, or even more, so one of the problems is that education and research and admin, they do not have the ability to recompense any physician, surgeons especially, the same way that clinical work does” [006-I-M-L].*

The discrepancy between compensation and workload in leadership roles identified by participants is a concern that relates to both the reward and fairness domains under the AoW model. Participants indicated that surgeons who are invested in the success of the department take on leadership or service roles only to receive more work in return. While certain positions may come with a small stipend, the increased income is not equivalent to the money lost from the necessary reduction in clinical tasks. It was conveyed that surgeons who put effort into improving the department are financially worse off than surgeons who are self-interested and focus primarily on clinical work. Participants expressed frustration that those who neglect the educational or administrative duties inherent to academic surgery are rewarded rather than facing repercussions for prioritizing their needs over the collective good of the department. Furthermore, this leaves surgeons who are dedicated to education and leadership responsible for “picking up the slack” of those who disregard these expectations, thereby further increasing their workload and feelings of resentment toward other staff. Another surgeon, when asked if they were adequately rewarded for their service roles, stated,

*“Adequately? No, not adequately, no way. I mean, there are really good people that you kind of work with along the way, that’s a nice dynamic, but there is no way that they can ever adequately compensate you for your time or effort [...] The trouble is, again, with our system, there’s very few carrots to entice people into nonclinical work and there’s absolutely no stick, right, so it’s very hard to [...] distribute that work, especially if you do not feel a whole lot of guilt or responsibility to do it,” [009-I-M-L].*

It was also provided that the lack of reward for leadership positions contributions also insinuates that the efforts of current leaders are unappreciated. One participant described the negative relationship with their leadership roles by saying,

*“I’m stupid enough to take on additional jobs, research students and stuff like that [...] I’m also the director of [leadership position]; I have a big role with that. I also just took on [other leadership position] — really dumb. But, you know, again, [I have] a big role with that as well, and all of those things are problematic, especially for some of us that are still on fee-for-service*” *[003-I-M-L].*

Participants asserted that receiving verbal recognition or praise for their work would not alleviate their burnout because the exhaustion associated with their daily tasks would remain. However, it was suggested that an alternative funding plan may provide incentive for more surgeons to be involved in administrative and leadership roles, potentially resulting in a more equitable distribution of work and greater sense of solidarity within the department.

One surgeon shared their thoughts on the impact that an alternative funding plan, such as one that included a base salary, would have on the workload and wellbeing of surgeons:

“*I think that [an alternative funding plan] would certainly help a lot because then it frees up more time to be able to look after patients better, to be able to do more administrative stuff, academic stuff, research stuff that a lot of people want to do [...]. I think that also helps with the volume stuff, so if all of a sudden you are making a good salary, you know, you may say, ‘Well, [if] we pull somebody else on board, you know, I’m not going to lose a whole lot of money now, so why do not we just hire somebody else and they can do some of these patients for us too?’” [003-I-M-L].*

Participants shared the belief that moving away from a fee-for-service payment plan would reduce the pressure surgeons feel to work extremely demanding hours. Interestingly, some surgeons shared that they prefer the fee-for-service model, as it allows them to have more flexibility in the hours that they work. One participant expressed this in saying,

“*The one thing that’s really nice about fee-for-service is that it gives me the control, right? So if I want to take a morning off to drop my kids off at school or if I want to take an afternoon off because I’m just feeling really burnt out… right now, I take that financial hit and I deal with the repercussions,” [010-I-W-L].*

This perspective inadvertently illustrates another consequence of a pure fee-for-service model: surgeons do not have access to paid time off. While it can be argued that the fee-for-service model treats surgeons as self-employed and therefore responsible for managing their time off, the situation is complicated by the facts that surgeons are accountable for their patients whose issues may arise at unpredictable times, they do not have control over the income they receive per billable service, and there are additional non-clinical responsibilities associated with academic surgery. A lack of incentive — or, more accurately, a cost — to take time off effectively discourages surgeons from maintaining a healthy work schedule and obtaining adequate rest.

It is clear that a lack of reward for nonclinical tasks is impacting surgeons’ morale and contributing to dimensions of burnout. It can be argued that the most transparent way in which an institution communicates its values is through remuneration. The majority of the current payment plans within the department indicate that research, administrative work, and teaching are underappreciated. The consequences of these payment plans impact all of the AoW domains, and are therefore a critical avenue through which impactful change can be made. Responses from participants strongly suggest that reassessing the department’s values and ensuring that actions that align with those values are rewarded and adequately compensated will promote a healthier and more productive culture.

#### 3.2.3 Trenches and ivory towers: the divide between problems, solutions, leadership, and the frontline

Surgeons described a disconnect between leadership and the “front line,” a mismatch between chronic sources of stress and the proposed solutions, as well as tension between staff members themselves as being contributing factors to their ongoing feelings of frustration and burnout. This theme lies at the intersection of values, control, fairness, and community; while conflict between leadership and care providers raises the issue of competing values and control over one’s practice, issues between surgeons themselves and the individualistic culture prevalent in surgical departments prevent surgeons from forming a cohesive community with their colleagues and those in leadership positions.

The dissonance outlined by participants with respect to the conflict they experience with leaders lies at the division between surgeons holding the scalpel and administrators making the decisions around when and for what purpose the scalpel can be used. Many participants indicated that administrators often enforce policies that do not align with the needs and values of those at the frontline of surgical care, such as prioritizing the allocation of resources based on financial motivations over health system needs. When concerns over this disconnect are raised, they are often met with ambivalent attitudes or remain unacknowledged. One surgeon commented,

*“As I see it, the organization is quite fixed… they have their own viewpoints or set points and they enact or enforce change that does not resonate with us at the front line.. it’s always with the undertone of saving the capital budget”* [002-I-W].

Enforcement of policies which are misaligned with the views of the frontline also contributes to the lack of control surgeons described in theme 1. Participants stressed that the lack of action and ambivalent attitudes that are present when concerns are raised lead surgeons to feel disenfranchised, and fosters feelings of resentment, cynicism, and a loss of trust in those in administrative positions. Another surgeon commented:

“*I think having some elements of leadership that actually can see these issues [is important] and to be honest, I think a lot of the time they do see issues, the question is why are these things not acted on?” [001-I-W-L].*

Moreover, despite the widespread acknowledgement among participants that the causes of burnout are primarily systemic in nature, surgeons indicated that the solutions and remedies suggested to staff members are predominantly individual-based modalities such as mindfulness interventions and stress management techniques. Not only do these proposed “solutions” ignore precipitating factors of burnout for surgeons and contribute to feelings of self-blame, but this disconnect also reinforces cynical attitudes that sustained change is beyond reach, thereby further driving a wedge between administration and staff. Participants emphasized that surgeons do not require more skills to cope with their ongoing demands; rather, there needs to be ongoing evaluation of the resources required to support clinicians in providing quality patient care, and proactive strategies developed to address these issues at their root cause. The superficial and reactive “support” strategies that are currently in place are not only ineffective, but also foster discontent among staff which is counterproductive and further contributes to feelings of burnout. One surgeon commented,

“*In my humble opinion, if you do not change the environmental conditions that lead to burnout… there is no support that can handle that” [13-S-M-L].*

Participants also reported that this disconnect is prevalent among staff members themselves. Participants speculated that the individualistic culture present in surgical specialties leads many of their colleagues to characterize poor surgical outcomes and feelings of burnout (as well as mental health concerns more generally) as an indication of personal deficiencies rather than broader health system issues. This cultural perception of burnout as a moral failing has led to many of the participants being reluctant to share their concerns and seek support from colleagues and leadership regarding patient care and complications out of fear of reprisal and judgment. One participant shared,

*“Not infrequently such events [surgical complications] may become the subject of a hospital complaint, a CPSO [College of Physicians and Surgeons Ontario] complaint, or a medicolegal case and we are discouraged from discussing such matters outside of the investigation or process. There is also the reservation of sharing personal or professional matters with colleagues for fear of being identified as weak or unfit for practice in any way.” [012-S-M].*

Participants indicated that many of their colleagues glorify overwork and see those who seek support or share their challenges with others as not being cut out for the specialty. These attitudes prevent staff members from leaning on each other when complications arise and reinforce the individualistic culture and divide between staff members outlined in theme 2.

Ultimately, surgeons emphasized that a key component to tackling burnout is to establish robust communication avenues between staff and leadership at various levels of the university and department. Those at the front line of patient care require engaged and supportive leadership who acknowledge and respond to issues when they arise. Open communication and a willingness to understand the responsibilities and pressures surgeons face on a day-to-day basis are critical to addressing the issues at the source, and preventing feelings of resentment toward leadership that foster conditions of burnout. Moreover, it is crucial that individual-focused interventions not be the sole remediation strategy offered to those experiencing burnout. Not only are these interventions not supported as primary prevention efforts in the literature, but they also further feelings of cynicism and a “victim-blaming” mentality among staff members who locate the root cause of burnout within the individual. Efforts to reduce the stigma associated with this syndrome should also come with education regarding the role of environmental stressors in the emergence of burnout, as well as the importance of having a supportive community in the workplace that is free from judgment and incivility toward others.

#### 3.2.4 Exacerbating factors

While the previous themes outline conditions that participants described as precipitating factors for burnout, there were two notable circumstances that were identified as exacerbating conditions for the pre-existing issues contributing to burnout: (1) gendered expectations and gender inequity, and (2) the COVID-19 pandemic.

##### 3.2.4.1 Gendered expectations and gender inequity

Women interviewed in this study indicated that they are disproportionately impacted by burnout as they are often burdened by tasks and behavior that are known risk factors for this syndrome. They described being expected to be more flexible and understanding of being treated in ways that are inconsiderate of their time as well as being expected to perform tasks that men in the department are typically not expected to complete. These expectations contribute to feelings of cynicism and exhaustion congruent with burnout, and intersect with the workload, fairness, reward, and control domains of the AoW model.

Women in this study described experiencing an increased workload due to being expected to perform secretarial or administrative tasks, as well as being given more “emotional” patients from colleagues who require consolation and additional time for consultation. Not only do these tasks take away women’s time from their primary role as surgeons, but it also reinforces the gendered stereotype that secretarial and emotional labor should be performed by women instead of men even within the same profession. Additionally, the migration of these tasks inevitably contribute to women spending less of their time on billable procedures and more time on kinds of work that have been established as risk factors for burnout. One surgeon commented:

“*I think it’s a kind of— a little bit taboo to discuss… I know that the work that I do is often reimbursed at a lesser amount than the work that some of my colleagues are doing. And that can make you feel like your work or your contributions are not valued [...] I find it really frustrating that people say things like, ‘Well, it’s fee for service, so there is no gender pay gap — you are getting paid for what you are doing so does not that mean we are all being paid fairly?’ And I would try to explain the different patient expectations, the different expectations for colleagues, the different types of patients that are being sent to women, like referral bias. You know, the fact that procedures done by women are often reimbursed at a lower rate because most of the committees that determine reimbursement are stacked by men.” [010-I-W-L].*

In instances where women do not accommodate the additional workload associated with these tasks, they are perceived as being inflexible; thus, many choose to oblige these requests or learn to “say no nicely.” However, times in which women “choose” to accommodate such a workload are not necessarily reflective of a choice, but rather the pressures that women in this profession experience around not being seen as lesser than their counterparts who are men. Some women expressed that they are hesitant to discuss issues of gender equity due to the perceptions that it is not a serious issue by some of their colleagues, and to be seen as a “whiner” or in need of additional support would have negative implications for their careers. One participant stated:

*“…the fact that I’m a female and I can be pushed more to do everything with less [contributes to burnout]… the more that you complain [the more] people find you annoying and whiny and difficult…that’s kind of not fair because I’m pretty sure that a male in my position would not stand for this…” [001-I-W-L].*

The balance between professional and personal responsibilities was also discussed as a challenge that disproportionately impacts women in academic surgery. The expectations for surgeons who are mothers tend to be different from what their colleagues who are men experience due to social norms around parenting. Mothers are expected to be ever-present and the primary caretakers for their children, even in family situations in which their partners are responsible for such tasks. One participant described this frustration in saying:

*“I mean… It’s just different. It’s mothers who are expected, who get called, when the kid’s sick at school. It’s not my husband. We’re the [expected] primary caretakers. I’m not actually, my husband is.” [004-I-W-L].*

Those who participated in this study advocated for the department to promote an increased awareness and understanding of the pressures and biases women face in this career, and ensuring women are involved in decision making around workplace policies and departmental organization through leadership positions. These surgeons emphasized that they do not want to be “treated differently than the men,” but rather, seen as equally competent and deserving of respect for their time and skill as surgeons.

##### 3.2.4.2 The COVID-19 pandemic

Since its emergence, the COVID-19 pandemic has placed unprecedented stress on our healthcare system and availability of health resources. For surgeons, especially those whose cases are mainly elective procedures, the pandemic further limited operating time, thereby adding to the already seemingly never-ending waitlists of patients and increased feelings of guilt around not being able to provide the care that people require in a timely manner. The additional restrictions on operating time that were put in place due to surges in COVID-19 cases also introduced novel conditions of financial stress for those on fee for service payment plans. The extreme reduction in cases meant surgeons were unable to bill for their services, thereby dropping their incomes drastically. For some surgeons, the salary of their administrative staff and other overhead costs was equivalent to or greater than the income they were making from their reduced caseload, meaning they were losing money for substantial periods of time during the pandemic. Moreover, when public health mandates relaxed and the OR became more accessible, the time allotted to elective procedures remained significantly reduced, preventing surgeons from returning to their pre-pandemic caseloads. This shift in OR resources and time reduced payments by tens of thousands of dollars per month for some surgeons. One participant commented:

*“So I think generally, all around there’s more work being expected of the staff surgeons, and then if we get a look at [the pandemic] following that… the issue with COVID-19 is that the ORs [operating rooms] have been shut down. We’ve had three or four ramp downs now when the OR67s were completely closed, except for urgent or emergent work. And as fee-for-service physicians, that means you do not get any money.” [006-I-M-L].*

While the pandemic was framed as a contributing factor to burnout, surgeons also emphasized that the patient-related pressures being experienced from COVID-19 were largely a result of pre-existing issues in the healthcare system being exacerbated. The increasing expectations of patients and referring physicians were noted as contributors to the pressures faced by surgeons in their careers. One participant stated:

“*So it [patient-care related pressures] definitely existed pre-pandemic, but everything has been worsened because wait times are so much worse than they used to be. I also think that expectations of patients and of referring doctors has changed and become much more demanding.” [009-I-M].*

Increased wait-times due to the pandemic response also exacerbated burnout for surgeons by reducing the control they have over when they are able to treat patients, an issue explored in theme 1. This was described as being especially problematic for surgeons who primarily complete elective, quality-of-life based procedures which were deprioritized during the pandemic in favor of emergent cases. Additionally, surgeons acknowledged that the pandemic prevented individuals from seeking and accessing medical care, which exacerbated conditions of moral injury, both due to guilt regarding not being able to reach patients in need, and also in anticipation of poor outcomes that could have been prevented if treatment had been delivered earlier. One surgeon poignantly expressed this fear in saying,

“*They [patients] are going to be presenting with more advanced disease, and so we know that, you know, had we seen them six months, a year ago, we could’ve of cured them, now we cannot. So there’s going to be the potential for some moral injury there as well; for surgeons, for physicians, for the health care teams as they see people that they know, you know, without COVID, they probably could have been cured and now they cannot be cured.” [006-I-M-L].*

Ultimately, the COVID-19 pandemic was identified as a contributor to burnout, mainly through the exacerbation of pre-existing issues in the healthcare system. The pandemic introduced extreme, unexpected financial stress on surgeons paid through fee-for-service, magnifying the limitations of this model. It also contributed to moral injury surgeons were already experiencing due to a lack of control in their provision of patient care. While the pandemic is, of course, outside of an individual institution’s control, it is critical that a surgical department analyzes the concerns that have been exacerbated and uses the lessons the pandemic provides to improve surgeons’ wellbeing, both in times of crisis and normalcy.

## 4 Discussion

This study explored the experiences of surgeons with burnout at an academic medical center using an organizational lens to identify target areas and potential strategies for change. Ultimately, participants described the extreme pressures they face while attempting to manage high patient volumes with limited resources, and the consequential guilt and unfulfillment they experience when they are unable to provide the quality of care to which they aspire. The theme “The Red Queen’s Race” is a reference to Lewis Carroll’s, “Through the Looking Glass” (1872), in which the Red Queen tells Alice that in the new land she finds herself in, she must run as fast as she can just to stay in the same place (42). Since the book’s original publication, this term has been used to describe concepts in various subjects including biology and economics. Here it is used to describe the futility surgeons feel in response to their demanding clinical workload: while they work as much and as hard as they can, they can never seem to keep up with the unceasing influx of patients requiring care. This finding is consistent with previous research that has identified that one of the primary stressors impacting physician wellbeing is an inability to provide quality healthcare, one of the most fulfilling aspects of a surgeons’ work day (43, 44). Moreover, there have been links made between burnout in healthcare workers and moral injury, a phenomenon which refers to the adverse psychological response that occurs following events that conflict with one’s values and moral beliefs (45, 46). It should also be noted that burnout itself may create conditions in which moral injury is more likely to occur. Burnout has been linked to a risk of major medical errors and lower quality patient care due to the cognitive and emotional deficits that accompany this syndrome (47), potentially creating a reciprocal and iterative spiral in which surgeons experience more opportunities for moral injury, and subsequently burnout, to manifest.

It was also expressed that conflict between decisions made by hospital leadership and surgeon needs, as well as emphasis on individualistic rather than systemic solutions to burnout, has created an environment that is incompatible with facilitating a healthy, supportive work community. Participants reported that this uncollaborative and individualistic culture drives a sense of hopelessness and futility surrounding the possibility of improvements in wellbeing across the department. This finding highlights the incongruence between our theoretical understanding of burnout as a response to chronic workplace stressors and the individualized interventions offered to address this issue (11, 48); the interventions presented to surgeons (and those in healthcare more broadly) largely focus on solutions that focus on stress management and building individual resiliency to burnout (2, 49, 50). While these approaches have demonstrated short-term merit in some settings, the participants in this study indicated that the presentation of individualized interventions to systemic issues reinforces feelings of frustration and actually contributes to issues of self-blame, and a culture in which the individual is blamed for system-level issues. This finding is consistent with research focused on such interventions, and in alignment with research that has indicated medial professions in particular tend to promote inappropriate self-care by misplacing system-level failings on the individual (1, 2, 51, 52). When examining the issues that participants indicate are causing burnout, it becomes clear that individualized solutions are not enough to prevent the issues encountered by surgeons.

The presentation of individualized solutions may also be reinforcing counterproductive behaviors learned throughout medical training, as well as deeply ingrained professional identity characteristics that prevent physicians from prioritizing their own wellbeing (53). The professional identities of physicians and surgeons are shaped by cultural norms within medical institutions that emphasize ideals of invincibility, stoicism, and perfectionism (54, 55). The perceptions of medicine as a virtuous and noble calling further perpetuates the notion that extreme personal sacrifices are inherent to the profession, and that enduring adverse working conditions is necessary to fulfill the role of a physician (53). Acknowledging vulnerability is often perceived as a sign of weakness or incompetence, and adhering to these standards creates tension between being a human being and being a physician (56). These professional norms and the stigma associated with violating them both impedes worklife balance and prevents physicians from speaking about problems that cause suffering, making it even more critical that approaches to mitigating burnout focus on broader cultural and institutional influences.

The exacerbating factor of gender inequity impacted burnout through the AoWs of fairness and workload, as women in surgery described being expected to complete more administrative and emotion work than their colleagues who are men. The balance between parental responsibilities and a demanding career was also outlined as a particularly challenging aspect of being a woman in surgery; even in parental situations in which the surgeon’s partner is responsible for childcare, the assumption remains that women are the primary caretakers. The culmination of difficulties that women face in academic surgery has aptly been referred to as the “double-edged scalpel” (57). Surgery as a specialty has historically been dominated by men, and gender disparities continue to be prevalent in this field (58–60). Many of the challenges that these women encounter are difficult to characterize through traditional quantitative methods (57, 61); however, the biases described by women in this study, specifically those regarding pay inequities, parental norms and policies, increased administrative work, and referral biases, have received empirical support (43, 62–65). The hesitation that women in this study described to address issues of gender equity for fear of judgment has also been captured in relation to other issues regarding gender discrimination, such as instances of sexual harassment and assault (66). Despite these challenges, women in surgery have surgical outcomes that are equivalent or superior to those of their colleagues who are men (67, 68). However, this should not come as a justification for allowing such instances of injustice to persist; there is robust evidence linking issues of gender-equity to burnout, and adverse impacts on women’s careers and wellbeing. It is critical that these inequities be addressed through policy reform at both a national and institutional level to affect widespread change.

A clear foundational issue that underlies the generated themes is the current fee-for-service pay structure that is prevalent across surgical specialties. The fee-for-service model contributes to incongruencies in fairness, workload, reward, values, and control, making it an obvious target of interventions to reduce burnout. As non-clinical work is compensated far less than clinical cases, surgeons in this study who are involved in service roles described feeling as though their work is unappreciated and these roles are ultimately not worth the effort they require. This payment plan also contributes to a culture in which time off is not prioritized or accessible, thereby exacerbating the effect of high workloads on surgeon wellbeing. While the fee-for-service model in direct relation to burnout has not been explored thoroughly, its connection to increased documentation and workload, as well as a loss of autonomy and has been established (69–71). For example, physicians and health system leaders have shared that receiving compensation for “desktop medicine” such as responding to messages and charting would improve their wellbeing, with progression to value-based, salaried payment plans being presented as a solution to burnout (71). It has also been argued that remuneration is the way institutions communicate their values to staff, and therefore, payment plans must be informed by the values and goals of the institution (72). Within this framework, the present study would suggest that research, education, administrative work, and physician well-being are not currently being communicated as valuable. Interestingly, many of the surgeons interviewed shared that they continue to be involved in non-clinical roles despite the lack of compensation. This suggests that surgeons receive intrinsic reward from their involvement in these positions, and presents the possibility that increasing the extrinsic reward (specifically, tangible compensation) associated with these roles could improve the sense of fulfillment and worth surgeons feel as a result of their non-clinical work.

Unique to this research was the use of a qualitative case study grounded in a framework designed specifically to evaluate organization risk factors for burnout. To our knowledge, this is the first qualitative study to employ the AoW model as a theoretical tool to identify potential issues leading to burnout in academic surgery. This study demonstrates how this model can be applied in a rigorous manner to identify “unit-level” issues that are critical in the formation of interventions for addressing burnout. Furthermore, our methodological choices highlight the promise of qualitative methods in gaining insight into the issues underlying the emergence of burnout and as a mechanism for soliciting direct feedback from those impacted by this issue. While some of our findings may apply more to our local department than to others, we do not view this as an inherent limitation, but rather as an intentional methodological and theoretical choice. However, these results should be interpreted within the scope of the local context and in consideration of the unique characteristics of these surgeons; these results are reflective of the pressures faced by surgeons at a single academic medical center within the Ontario healthcare system. Future work will explore these potential contributors in more detail and strategies to address burnout within this institution.

## Data availability statement

Datasets presented in this article are not readily available due to the highly identifiable nature of the content, and participants’ requests to keep their data and experiences confidential. Questions about these datasets should be directed to KM, mcneillk@mcmaster.ca.

## Ethics statement

The studies involving humans were approved by Hamilton Integrated Research Ethics Board. The studies were conducted in accordance with the local legislation and institutional requirements. The ethics committee/institutional review board waived the requirement of written informed consent for participation from the participants or the participants' legal guardians/next of kin because of COVID-19 restrictions for in-person research. Obtaining written consent was not feasible for the research team or participants. Instead, implied and verbal consent was obtained for the virtual survey and interviews, respectively.

## Author contributions

KM: Conceptualization, Data curation, Formal analysis, Funding acquisition, Investigation, Methodology, Writing – original draft, Writing – review & editing. SV: Data curation, Formal analysis, Writing – original draft, Writing – review & editing. SC: Data curation, Formal analysis, Writing - review and editing. IY: Conceptualization, Funding acquisition, Supervision, Writing – original draft, Writing – review & editing. RS: Conceptualization, Funding acquisition, Supervision, Writing – original draft, Writing – review & editing.
