# Supplementary material for: “I don’t know if I can keep doing this”: a qualitative investigation of surgeon burnout and opportunities for organization-level improvement
Source: Front Public Health. 2024 May 10;12:1379280. doi: 10.3389/fpubh.2024.1379280 (PMC11116672; doi:10.3389/fpubh.2024.1379280)
Supplement: Supplementary file 1 [file Data_Sheet_1.docx]

Supplementary Material

# Survey Questions

How do you think that burnout has manifested in your personal and professional lives?

Are there any specific areas that you think the department, administration, or your colleagues could improve on that might mitigate or alleviate feelings of burnout?

Do you feel as though you can access adequate support through your department for work-related impact on your mental wellbeing? Why or why not?

Is there anything else that you would like to tell us regarding burnout in your workplace?

# Interview Questions

How do you think that burnout has manifested in your personal and professional lives?

How has burnout influenced the choices and changes you’ve made throughout your career and personal life?

Are there any tasks or specific demands associated with your work that are particularly exhausting and that you feel contribute to burnout?

Do you feel as though you have adequate opportunity to recover from strenuous workloads and feelings of burnout, and is there anything that might aid this recovery that you don’t currently have access to?

Do you feel as though you’re able to access all of the resources needed for you to thrive in your workplace and is there anything you’re not able to access that might make your job easier or mitigate feelings of burnout?

Does the level of responsibility or control you hold over your work contribute to feelings of burnout and might a change in the level of the responsibility or control you have over your work mitigate these feelings?

Do you feel as though you are adequately rewarded and receive recognition for the contributions you make in your work and do you think here’s a link between how you are rewarded or the amount of recognition you receive and feelings of burnout?

In what areas do you feel surgeons in this Department may benefit from more recognition, and is there anything that the Department or your colleagues might be able to contribute?

Do you feel as though you have a community in your workplace in which you’re able to find support?

Are there any specific areas that you think the department, administration, or your colleagues could improve on in terms of communication, cooperation, or social support that might mitigate or alleviate feelings of burnout?

Do you feel as though there are decisions concerning your work, such as promotional decisions and allocation of resources, that may be unfair and contribute to burnout?

Do you think that your department can effectively resolve issues of unfairness and are there any areas that the department can work on to promote fairness in the workplace?

What kinds of values related to your work did you have prior to entering surgery and how have these values changed over the course of your career?

Are there any areas of your work or aspects of healthcare/surgery that conflict with your personal values, and do you think this conflict contributes to burnout?

Are there any areas that we haven’t discussed that you feel might contribute to burnout in your workplace or any other suggestions that you have for the department for strategies to mitigate burnout?

Is there anything else you would like to add?
